# Purification, Preliminary Characterization, and Immunological Activity of Polysaccharides from Crude Drugs of Sijunzi Formula

**DOI:** 10.1155/2017/2170258

**Published:** 2017-07-16

**Authors:** Yunfei Ji, Ruijun Wang, Ying Peng, Chongsheng Peng, Xiaobo Li

**Affiliations:** School of Pharmacy, Shanghai Jiao Tong University, Shanghai 200240, China

## Abstract

Sijunzi Decoction (SJZD) is a conventional prescription for curing spleen deficiency in Traditional Chinese Medicine and polysaccharide is its main ingredient. In order to explore the effective ingredients contributing to the immunological activity of SJZD, we isolated and purified seven homogeneous polysaccharides from Radix Ginseng (RS-3-1 and RS-3-2), Rhizoma Atractylodis Macrocephalae (BZ-3-1, BZ-3-2, and BZ-3-3), Poria (FL-3-1), and Radix Glycyrrhizae (GC-3-1) decoctions, respectively. The molecular weight of seven homogeneous polysaccharides ranged from 5.42 × 10^4^ to 5.65 × 10^4^ Da. Monosaccharide composition determined by GC-MS analysis showed that these polysaccharides were primarily composed of Rha, Ara, Xyl, Man, Glc, and Gal with various ratios. Immunological activity assay revealed that polysaccharides from four crude drug components of SJZD displayed inhibitory effects on the complement system. RS-3-1, BZ-3-1, FL-3-1, and GC-3-1 could significantly enhance the phagocytosis and increase the NO production and tumor necrosis factor (TNF-*α*) level in RAW 264.7 cells (*p* < 0.05). These results demonstrated the immunological activities of these polysaccharides from the four crude drugs. This study supports the therapeutic effect of SJZD in clinical use and is essential for further identification the immunopolysaccharide from SJZD decoction.

## 1. Introduction

Sijunzi Decoction (SJZD), a conventional Traditional Chinese Medicine Formula (TCMF), consists of four crude drugs: Radix Ginseng, Rhizoma Atractylodis Macrocephalae, Poria, and Radix Glycyrrhizae in the ratio of 9 : 9 : 9 : 6. For thousands of years, it has been used to nourish Qi to invigorate the spleen of patients in China. Modern pharmacological studies have shown that Sijunzi Decoction can potentially revitalize the gastrointestinal function, strengthen the immune system, and be used as adjuvant therapy for cancers [1–4].

It was reported that polysaccharide from SJZD could affect the cellular migration and gene expression in wounded rat intestinal epithelial cells [5] and restore the intestinal flora to regulate immune function in spleen-deficient mice [6]. In a previous study, we used a comprehensive method, which assessed immune system regulation, intestinal microbiota, and short chain fatty acids, to screen for the active polysaccharide fraction from the SJZD with reserpine-induced rats. The results consistently indicated that the polysaccharide fraction S-3 from SJZD could enhance the immune function and treat spleen deficiency [7]. Furthermore, we isolated and purified two homogeneous polysaccharides from S-3 fraction (S-3-1 and S-3-2), and we found that S-3-1 could significantly enhance the anticomplement activity and macrophage immunological activity in vitro. However, little information is available about the constitution and source of this polysaccharide from SJZD.

Literatures have also reported multiple bioactivities of polysaccharides from crude drug components of Sijunzi formula. A water-soluble ginseng marc polysaccharide (GMP) was shown to be an effective immunomodulatory ingredient with the ability to stimulate the production of reactive oxygen intermediates [8]. Polysaccharide from Atractylodes Macrocephalae Rhizoma was known to regulate the gut microbes and is widely used in the treatment of chronic intestinal diseases [9]. A carboxymethylated-sulfated *β*-(1→3)-D-glucan extracted from* Poria cocos* (CS-PCS3-II) could significantly improve the increase the indexes of phagocyte, thymus, and spleen and promote spleen antibody production, hemolytic activity, and delayed-type hypersensitivity (DTH), suggesting the immunopotentiation of CS-PCS3-II in mice [10]. Radix Glycyrrhizae polysaccharide (GP) was demonstrated to decrease the mRNA expression of Foxp3 and IL-10 and upregulate Th1/Th2 cytokine ratio in serum in tumor bearing mice, which putatively inhibited tumor growth [11]. TCM prescription is usually a formula of several single herbs that are combined at a fixed ratio and boiled in water to form the decoction. Each herb has its own active components, but when multiple herbs are decocted together, the possible chemical reactions may result in new components that are biologically active. It is not clear that the active polysaccharides of SJZD are from the existing ingredients of the crude drugs or from the decocting process. Therefore, identification of the source of the active polysaccharides of SJZD is required.

In order to identify the source of the immunopolysaccharide from SJZD, we investigate preliminary characterization and immunomodulatory effects of polysaccharides isolated from four crude drugs of SJZD in the present study.

## 2. Materials and Methods

### 2.1. Materials

Radix Ginseng, Rhizoma Atractylodis Macrocephalae, Poria, and Radix Glycyrrhizae were purchased from Shanghai Huayu Pharmaceutical Co., Ltd. (Shanghai, China). The botanical origins were identified by the corresponding author, and voucher specimens were deposited at the School of Pharmacy, Shanghai Jiao Tong University. Trifluoroacetic acid (TFA), dextrans, monosaccharide standards, dimethyl sulfoxide (DMSO), and lipopolysaccharide (LPS) were purchased from Aladdin Reagent Int. (Shanghai, China). DEAE-Cellulose 52 and Sephadex G-100 were purchased from Solarbio Science & Technology Co., Ltd. All other chemicals used in this study were of analytical grade.

Mouse macrophage cell line RAW264.7 was obtained from the Institute of Cell Biology, Chinese Academy Sciences (Shanghai, China).

### 2.2. Extraction, Isolation, and Purification of Polysaccharides

SJZD polysaccharides were prepared as previously described [7]. Dried Radix Ginseng (Ren Shen), Rhizoma Atractylodis Macrocephalae (Bai Zhu), Poria (Fu Lin), and Radix Glycyrrhizae (Gan Cao) (200 g) were soaked in 2 L distilled water for 2 h and then kept boiling for 30 min twice. The extracts were combined and concentrated by rotary evaporator to 1 g/mL. The extracts were then added with four times volume of pure ethanol to precipitate the crude polysaccharide at 4°C overnight. The precipitates were collected by centrifugation and deproteinized by the Sevag method [12]. Then the polysaccharides were washed with pure ethanol, acetone, and ethyl ether sequentially. Finally, the crude polysaccharides were dissolved in distilled water and lyophilized. The crude polysaccharides of RS (Radix Ginseng, Ren Shen), BZ (Rhizoma Atractylodis Macrocephalae, Bai Zhu), FL (Poria, Fu Lin), and GC (Radix Glycyrrhizae, Gan Cao) were obtained.

300 mg crude polysaccharides (RS, BZ, FL, and GC) were dissolved in distilled water and loaded onto a DEAE-52 cellulose anion exchange chromatography column (26 mm × 60 cm), respectively, which was sequentially eluted with 0–0.2 M NaCl, 0.3 M NaCl at a flow rate of 0.5 mL/min. The 0.3 M NaCl elution of each single herb's crude polysaccharides was collected and named individually (RS-3, BZ-3, FL-3, and GC-3). Then the carbohydrate contents of four crude polysaccharides fractions were assessed by phenol-sulfuric acid method, further purified by Sephadex G-100 size-exclusion chromatography column (26 mm × 70 cm), and eluted with deionized water at flow rate 0.25 mL/min. The seven polysaccharides obtained from the four herbs were named RS-3-1, RS-3-2; BZ-3-1, BZ-3-2, BZ-3-3; FL-3-1; GC-3-1, respectively.

### 2.3. General Analysis of Polysaccharides

Total carbohydrates were quantified by the phenol-sulfuric acid colorimetric methods [13], using D-glucose as the standard. Gel permeation and anion exchange chromatography were monitored by measuring the total sugar content. Total protein level was determined by Bradford method using BSA as the standard [14]. The content of uronic acid was measured by metahydroxydiphenyl method using D-galacturonic acid as the standard [15].

### 2.4. Determination of Homogeneity and Relative Molecular Weight

The homogeneity and molecular weights of RS-3-1, RS-3-2, BZ-3-1, BZ-3-2, BZ-3-3, FL-3-1, and GC-3-1 were evaluated and determined by high performance gel permeation chromatography (HPGPC) on a TSK-GEL G4000PWxl column (7.5 × 300 mm, Tosoh Co., Japan). The columns were maintained at 30°C, and the mobile phase was deionized water at a flow of 1 mL/min. T-series dextrans with different molecular weights (1 × 10^4^, 2 × 10^4^, 4 × 10^4^, 7 × 10^4^, 1.1 × 10^5^ Da) were used for the calibration curve. A 20 *μ*L aliquot was injected for each run. A standard linear curve was calibrated with dextran standards, *Y* = −0.0198*X* + 4.8426 (*R*^2^ = 0.9900). The molecular weight of these polysaccharides were estimated using the calibration curve equation.

### 2.5. Monosaccharide Composition Analysis

The monosaccharide composition was analyzed as described previously [16]. The hydrolyzed and acetylated derivatives of polysaccharides were loaded onto GC-MS (model 7890/5975C-GC/MSD, Agilent Technologies; Santa Clara, CA, USA). Each sample (5 mg) was hydrolyzed with 2 mol/L trifluoroacetic acid at 100°C for 2 h. The excessive acid was removed by evaporation with methanol for 3 times. For GC-MS analysis, the complete hydrolysate was mixed with 10 mg hydroxylamine hydrochloride and 0.5 mL pyridine and incubated at 90°C for 30 min. The mixture was cooled to room temperature and then incubated with acetic anhydride (0.5 mL) at 90°C for 30 min, and the resulting aldononitrile acetate derivatives were analyzed by GC-MS with inositol (0.5 mg) used as internal standard. The monosaccharide composition of RS-3-1, RS-3-2, BZ-3-1, BZ-3-2, BZ-3-3, FL-3-1, and GC-3-1 was determined based on retention time of six monosaccharide standards (L-rhamnose, L-arabinose, D-xylose, D-mannose, D-glucose, and D-galactose).

### 2.6. Anticomplementary Activity through the Classical Pathway

According to the method described by Jiang et al., the inhibitory effect of each polysaccharide fraction on activation of human complement through the classical pathway was examined by hemolytic assay [17]. 2% sheep erythrocytes with hemolysin were used on the same day. Guinea pig serum was used as the complement source. 1 in 20 dilutions of guinea pig serum was used to generate submaximal lysis of the antibody-sensitized sheep erythrocytes. In brief, various dilutions of polysaccharide samples (50 *μ*L) were mixed with 50 *μ*L of guinea pig serum, sensitized sheep erythrocytes (100 *μ*L), and BBS (100 *μ*L). The mixture was incubated at 37°C for 1 h. Inhibition of lysis (%) = 100 − 100 × (OD_sample_ − OD_sample  control_)/OD_100%  lysis_. The concentration showed that 50% hemolytic inhibition was defined as the CH_50_ value toward the classical pathway.

### 2.7. Macrophage Cell Immunological Activity

RAW264.7 cells were cultured in DMEM supplemented with 10% FBS at 37°C under humidified incubator with 5% CO_2_ [18].

#### 2.7.1. Assay of Macrophage Phagocytosis

The phagocytic ability of macrophages was determined by neutral red uptake [18, 19]. RAW264.7 cells were seeded in a 96-well plate (1 × 10^4^ cells/well) and treated with serial dilutions of RS-3-1, BZ-3-1, FL-3-1, GC-3-1 (1, 10, 50, 100, and 500 *μ*g/mL), DMEM medium, and LPS (5 *μ*g/mL) for 24 h. Then the cells were incubated with 0.075% neutral red solution (100 *μ*L/well) for 0.5 h. The cells were washed with PBS three times and incubated with cell lysis buffer (ethanol : glacial acetic acid = 1 : 1, 150 *μ*L/well) for 1 h. The absorbance was determined using a microplate reader (BioTek) at 540 nm wavelength. Phagocytosis index was calculated by the following equation:

(1) $$\begin{array}{c} Phagocytosis\;\;index=\frac{{Abs}_{\left(sample\right)}}{{Abs}_{\left(blank\;\;control\right)}}. \end{array}$$

#### 2.7.2. Assay of NO Production

Nitrite accumulation was measured using Griess reagent [18, 20]. RAW264.7 cells were seeded into 96-well plates (1 × 10^4^ cells/well) and stimulated with DMEM medium, LPS (5 *μ*g/mL), and various concentrations of polysaccharide samples (1, 10, 50, 100, and 500 *μ*g/mL) for 24 h. After incubation, 100 *μ*L of culture supernatants was transferred to another 96-well plate and incubated with Griess reagent as described in the manual. Nitrite concentrations in culture supernatants were measured to assess NO production in RAW264.7 cells:

(2) ConcentrationNO=Abssample−Absblank  controlAbsNaNO2−Absblank  control×ConcentrationNaNO2×Dilution  ratio.

#### 2.7.3. Quantitative Analysis of Cytokines

RAW264.7 cells (1 × 10^4^ cells/well) were seeded in 24-well plates and incubated overnight. Serial dilutions of polysaccharide samples (1, 10, 50, 100, and 500 *μ*g/mL), DMEM medium and LPS (5 *μ*g/mL) were then added, and the cells were incubated for another 24 h. The supernatants were collected and analyzed using an ELISA kit according to the manufacturer's instructions to determine the level of TNF-*α* secretion [21].

## 3. Results

### 3.1. Chemical Characterization of the Homogeneous Polysaccharides

**Figure 1:**
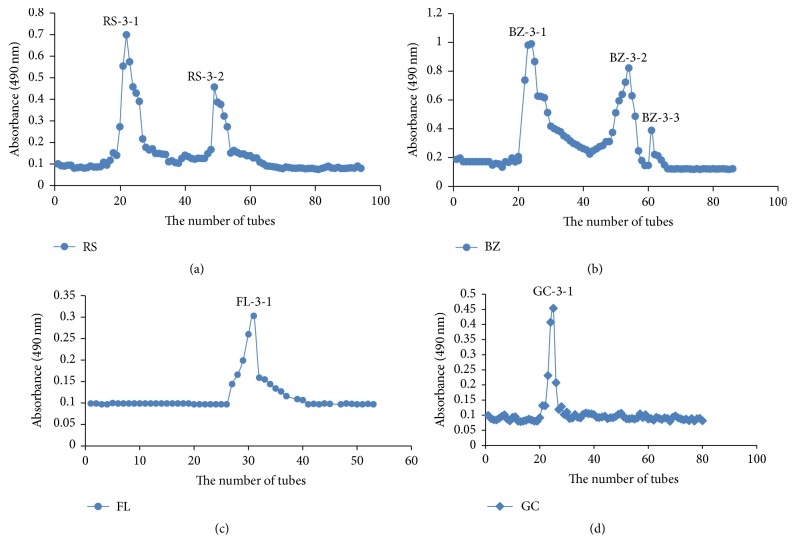
Elution curve of RS-3, BZ-3, FL-3, and GC-3 on Sephadex G-100 column to obtain RS-3-1, RS-3-2; BZ-3-1, BZ-3-2, BZ-3-3; FL-3-1; and GC-3-1: (a) RS-3: the 0.3 M NaCl eluted fraction of RS crude polysaccharide; (b) BZ-3: the 0.3 M NaCl eluted fraction of BZ crude polysaccharide; (c) FL-3: the 0.3 M NaCl eluted fraction of FL crude polysaccharide; (d) GC-3: the 0.3 M NaCl eluted fraction of GC crude polysaccharide.

Crude polysaccharides RS, BZ, FL, and GC were obtained from Radix Ginseng, Rhizoma Atractylodis Macrocephalae, Poria, and Radix Glycyrrhizae through water extraction, ethanol precipitation, and deproteinization and were further separated by DEAE-52 ion-exchange chromatography and Sephadex G-100 size-exclusion chromatography. As a result, two polysaccharides were isolated from Radix Ginseng (RS-3-1 and RS-3-2), three from Rhizoma Atractylodis Macrocephalae (BZ-3-1, BZ-3-2 and BZ-3-3), and one each from Poria (FL-3-1) and Radix Glycyrrhizae (GC-3-1) (Figure 1). Protein was not detected in these polysaccharides and the content of uronic acid was low. The HPGPC profiles suggested that the polysaccharides were homogeneous as each polysaccharide showed a symmetrical peak. According to the retention time, the average molecular weight was estimated as 5.57 × 10^4^, 5.44 × 10^4^, 5.65 × 10^4^, 5.59 × 10^4^, 5.51 × 10^4^, 5.58 × 10^4^, and 5.42 × 10^4^ Da, for RS-3-1, RS-3-2, BZ-3-1, BZ-3-2, BZ-3-3, FL-3-1, and GC-3-1, respectively (Table 1). The identity of isolated polysaccharides from the four crude drugs has not been documented in previous literatures.

### 3.2. Analysis of Monosaccharide Composition of the Polysaccharides

The monosaccharide composition of each polysaccharide was determined using GC-MS and the results are shown in Table 2. The results showed that RS-3-1 and RS-3-2 had different monosaccharide composition although they were isolated from the same native fraction RS-3. BZ-3-1 and BZ-3-2 contained the same types of monosaccharides (Rha, Ara, Man, Glc, and Gal), but the amount of Glc was higher in BZ-3-2. The monosaccharide composition of BZ-3-3 was different from the other two (BZ-3-1 and BZ-3-2). FL-3-1 was composed of Rha, Ara, Fuc, Man, Glc, and Gal in the ratio (molar) of 0.01 : 0.027 : 0.04 : 0.14 : 0.93 : 0.15. The monosaccharide composition of GC-3-1 was Rha, Ara, Man, Glc, and Gal, in molar ratio of 0.01 : 0.019 : 0.12 : 0.58 : 0.025. These results suggested that polysaccharides from different herbs varied dramatically in molecular weight and monosaccharide composition, and these structural features might be relevant to the biological activities. It could justify the inclusion and the ratio of the four herbs in this formula.

### 3.3. Anticomplementary Activity of the Polysaccharides

**Figure 2:**
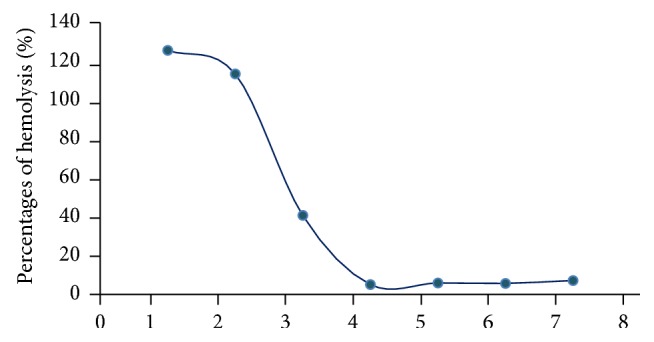
Percentages of hemolysis for different complement concentration: 1, 1 : 10; 2, 1 : 20; 3, 1 : 40; 4, 1 : 80; 5, 1 : 160; 6, 1 : 320; 7, 1 : 640 dilution.

**Figure 3:**
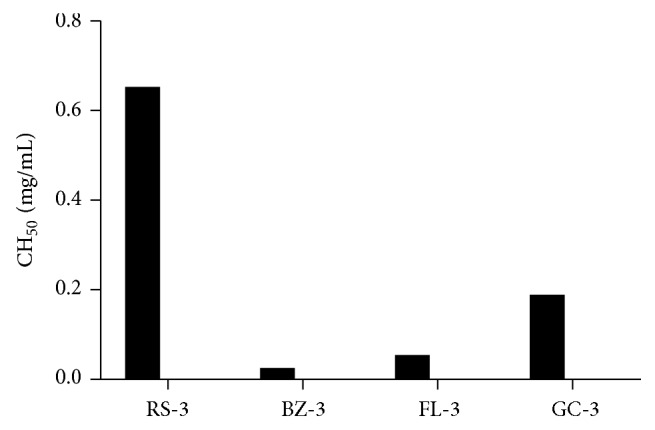
Anticomplement activities of the polysaccharide fractions obtained by DEAE-52 ion-exchange column from RS, BZ, FL, and GC: RS-3: the 0.3 M NaCl eluted fraction of RS crude polysaccharide; BZ-3: the 0.3 M NaCl eluted fraction of BZ crude polysaccharide; FL-3: the 0.3 M NaCl eluted fraction of FL crude polysaccharide; GC-3: the 0.3 M NaCl eluted fraction of GC crude polysaccharide.

**Figure 4:**
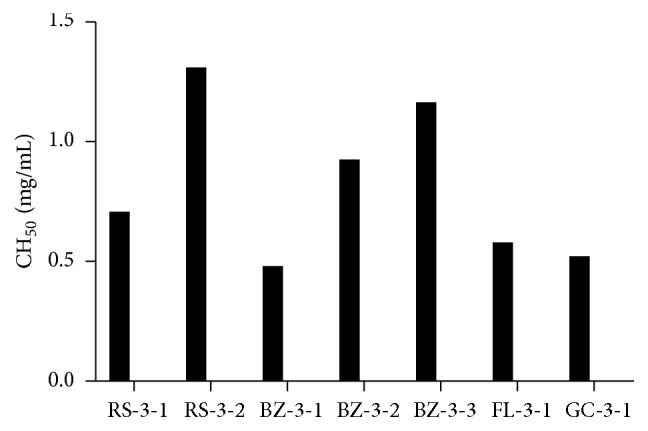
Anticomplement activities of the purified polysaccharides obtained by Sephadex G-100 size-exclusion column from RS-3, BZ-3, FL-3, and GC-3. (RS-3-1, RS-3-2, BZ-3-1, BZ-3-1, BZ-3-3, FL-3-1, and GC-3-1: the homogeneous polysaccharides obtained by Sephadex G-100 column from RS-3, BZ-3, FL-3, and GC-3 separately.)

As shown in Figure 2, the 1 : 20 guinea pig serum dilution induced almost 100% lysis of the antibody-sensitized sheep erythrocytes and was then selected as the complement source of the hemolytic assay. The inhibitory effects of each polysaccharide's fractions and homogeneous polysaccharides on human complement activation through the classical pathway were examined. The polysaccharide fractions isolated from DEAE-52 ion-exchange chromatography (RS-3, BZ-3, FL-3, and GC-3) all showed potent complement fixation activities as shown in Figure 3. The results of homogeneous polysaccharides (the further purified polysaccharides from Sephadex G-100 size-exclusion chromatography column) are shown in Figure 4. BZ-3, isolated by DEAE-52 column from Rhizoma Atractylodis Macrocephalae, showed the best anticomplement activity among the four polysaccharide fractions (CH_50_ 26.06 *μ*g/mL). Among the further purified homogeneous polysaccharides, BZ-3-1 showed better anticomplement activity.

RS-3-1, BZ-3-1, FL-3-1, and GC-3-1 were further chosen for the macrophage immunological study, as they showed better anticomplementary activities and their yields were abundant.

### 3.4. The Immunological Activity of Homogeneous Polysaccharides RS-3-1, BZ-3-1, FL-3-1, and GC-3-1

#### 3.4.1. Effects on Proliferation of RAW264.7 Cells

MTT assay was used to determine the effects on the proliferation of RAW 264.7 cells. No cytotoxicity was observed at any concentration to RAW264.7 cells after 24-hour incubation. Therefore, effects on representative macrophage functions, that is, phagocytosis, NO production, and cytokine production, were compared at 1, 10, 50, 100, and 500 *μ*g/mL among polysaccharides in RAW 264.7 cells.

#### 3.4.2. Phagocytic Activities

**Figure 5:**
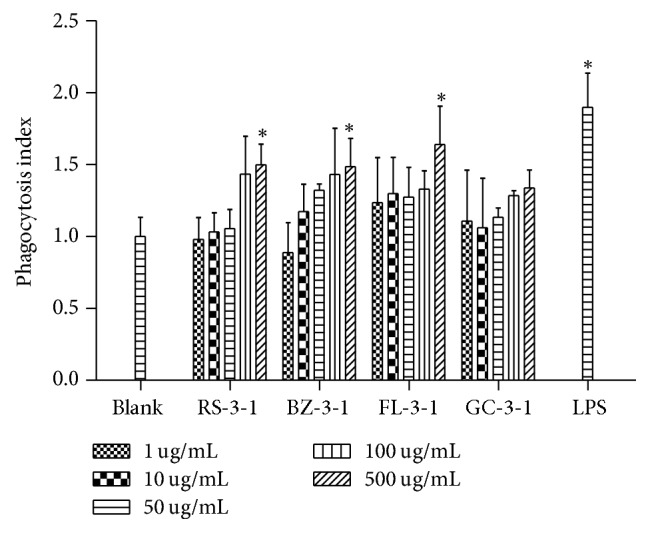
Effects of RS-3-1, BZ-3-1, FL-3-1, and GC-3-1 on phagocytosis activity of RAW264.7 cells. (Adherent RAW264.7 macrophages were incubated with various concentrations of polysaccharides (1, 10, 50, 100, and 500 *μ*g/mL) for 24 h. The DMEM medium and LPS (5 *μ*g/mL) were used as blank and positive controls, resp. ^*∗*^*p* < 0.05 compared with blank control).

The phagocytic activity is one of the most important functions of macrophages in innate immune responses [22]. The effects of RS-3-1, BZ-3-1, FL-3-1, and GC-3-1 on phagocytic activity of RAW264.7 cells were assessed by neutral red assay. As shown in Figure 5, after 24 hours of treatment, the phagocytosis index was elevated in a dose-dependent manner, by each polysaccharide except 1 *μ*g/ml of BZ-3-1. The stimulation of phagocytosis index induced by 500 *μ*g/mL of RS-3-1, BZ-3-1, and FL-3-1 was statistically significant compared to control group and was at a similar level to the LPS-treated group. These results indicated that RS-3-1, BZ-3-1, and FL-3-1 could enhance the phagocytosis of the macrophages, potentially through the binding of the polysaccharide to a specific receptor on macrophage cell surface.

#### 3.4.3. Effects on NO Production of Macrophages

**Figure 6:**
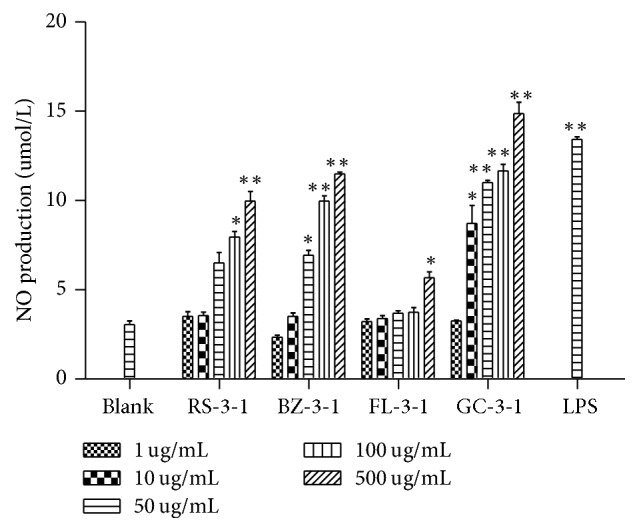
Effects of RS-3-1, BZ-3-1, FL-3-1, and GC-3-1 on NO production of RAW264.7 cells. (Measurement of NO release from macrophages after 24 h stimulation with various concentrations of polysaccharides (1, 10, 50, 100, and 500 *μ*g/mL) for 24 h. The DMEM medium and LPS (5 *μ*g/mL) were used as blank and positive controls, resp. ^*∗*^*p* < 0.05, ^*∗∗*^*p* < 0.01 compared with blank control).

The effects of RS-3-1, BZ-3-1, FL-3-1, and GC-3-1 on the NO production of RAW264.7 cells were determined by Griess assay. As shown in Figure 6, RS-3-1, BZ-3-1, FL-3-1, and GC-3-1 could significantly stimulate macrophages to produce higher levels of NO in a dose-dependent manner (*p* < 0.05). Furthermore, 500 *μ*g/mL of GC-3-1 showed stronger effect than the positive control LPS. These results suggested that RS-3-1, BZ-3-1, FL-3-1, and GC-3-1 can activate macrophages.

#### 3.4.4. Effects on TNF-*α* Production of Macrophages

**Figure 7:**
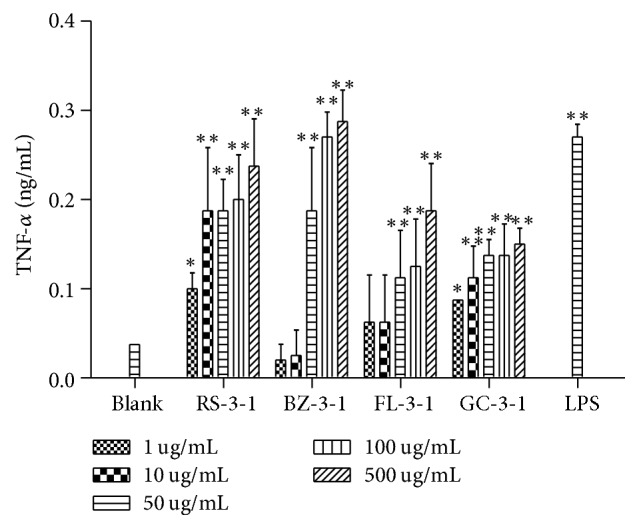
Effects of RS-3-1, BZ-3-1, FL-3-1, and GC-3-1 on TNF-*α* production of RAW264.7 cells. (Adherent RAW264.7 macrophages were incubated with various concentrations of polysaccharides (1, 10, 50, 100, and 500 *μ*g/mL) for 24 h. The DMEM medium and LPS (5 *μ*g/mL) were used as blank and positive controls, resp. ^*∗*^*p* < 0.05, ^*∗∗*^*p* < 0.01 compared with blank control).

As shown in Figure 7, RS-3-1, BZ-3-1, FL-3-1, and GC-3-1 showed significant stimulating effects on TNF-*α* production compared with the blank control group (*p* < 0.05), at all concentrations except 1 and 10 *μ*g/mL of BZ-3-1. Particularly, RS-3-1 showed a dose-dependent manner on TNF- *α* stimulation and approached the effect of LPS at 500 *μ*g/mL.

## 4. Discussion and Conclusion

We obtained seven homogeneous polysaccharides from four crude drugs (Radix Ginseng, Rhizoma Atractylodis Macrocephalae, Poria, and Radix Glycyrrhizae) of SJZD, using the same preparation method of S-3-1 (an active homogeneous polysaccharide from SJZD prescription we found previously [7]). Possibility of redundant polysaccharides was excluded by comparing the molecular weight, monosaccharide composition, and ratio with S-3-1. It was implied that the active polysaccharide S-3-1 could be formed during the decocting process by the sugar chain repolymerization. Research on polysaccharides of four crude drugs that comprise Sijunzi formula can assist in the identification of the source of immunopolysaccharide in SJZD.

In this study, the seven polysaccharides we isolated were all homogeneous polysaccharide with molecular weight ranging from 5.42 × 10^4^ to 5.65 × 10^4^ Da. In the previous study, one polysaccharide (PAM) which weights 2.88 × 10^4^ Da was obtained from Rhizoma Atractylodis Macrocephalae through the sequential water extraction, ethanol precipitation, and deproteinization method [9]. Additionally, one crude polysaccharide (WGPA) was also obtained from Radix Ginseng, and six acidic fractions (WGPA-1-RG, WGPA-2-RG, WGPA-1-HG, WGPA-2-HG, WGPA-3-HG, and WGPA-4-HG) were further purified by a combination of ion-exchange and gel permeation chromatography [23]. The molecular weights of these six polysaccharides ranged from 3.5 × 10^3^ to 1.1 × 10^5^ Da. The different process of isolation and purification may result in different polysaccharide extracts from the same herb. In above references, the polysaccharides were obtained using the DEAE-Cellulose column eluted with a linear gradient from 0.0 to 1.0 M NaCl, plus a Sepharose CL-6B column eluted with 0.15 M NaCl. However, in our study, we adopted the combination of DEAE-52 ion-exchange and Sephadex G-100 size-exclusion chromatography firstly in the purification of the four crude drugs.

The complement system is an important part of the innate immune system which also cooperates with the adaptive immune system in many ways. The complement system participates in the immune response and is involved in a variety of infectious and noninfectious inflammatory diseases. Therefore, appropriate inhibition of excessive activation of the complement system is important in immunomodulatory pathways. The complement fixation assay has been used for a long time as a screening system for the interaction with the human immune system [24–27]. In the present study, anticomplementary assay of the polysaccharide showed that the seven homogeneous polysaccharide had potential anticomplement activity. It has been reported that polysaccharides with higher molecular weights appear to be more active in the complement assay [28]. Among the seven homogeneous polysaccharides identified in our study, BZ-3-1, the one with the highest molecular weight, indeed exhibited the highest anticomplement activity.

Activation of macrophages can enhance their antitumor activity through secretion of cytokines or regulation of the immune system [29]; thereby, it is commonly assessed to examine the immunomodulatory function of polysaccharides [21, 30]. In this study, macrophage immunological activity assay revealed that RS-3-1, BZ-3-1, FL-3-1, and GC-3-1 could enhance phagocytosis, promote NO production, and increase tumor necrosis factor (TNF-*α*) level in RAW 264.7 cells. It has been reported that Radix Glycyrrhizae polysaccharide (GP) extracted from Radix Glycyrrhizae could modulate macrophage immune functions, induce nitric oxide (NO) production and NO synthase (iNOS) transcription, and reduce oxidative stress in mice [31, 32]. Glc was the only identified monosaccharide composition of GP, which is different from that of GC-3-1 in our study. The derived polysaccharide from the same herb might be the key for macrophage activation. The arabinogalactan side chains of WGPA-2-RG, A type I rhamnogalacturonan (RG-I) pectin obtained from ginseng polysaccharides, have been found to be the essential structure for stimulating macrophage phagocytosis and lymphocyte proliferation [33]. RS-3-1 containing monosaccharide Ara may be the critical factor of the effect on macrophage cells. In the present study, the effects on phagocytic activity are ranked as RS-3-1 < GC-3-1 < FL-3-1 < BZ-3-1; the ranking for NO production was FL-3-1 < RS-3-1 < BZ-3-1 < GC- 3-1 and FL-3-1 < GC-3-1 < RS-3-1 < BZ-3-1 for TNF-*α* secretion. Given that BZ-3-1 was the most active polysaccharide obtained from the single herb and it showed similar monosaccharide compositions to S-3-1, it is likely that BZ-3-1 is the source of active immunopolysaccharide from SJZD. Therefore, further characterization of its structure will be required.

In conclusion, we investigated the polysaccharides isolated from four crude drugs that comprise SJZD, and the result supports the therapeutic effect of SJZD in clinical use and is essential for further identification the immunopolysaccharide from SJZD decoction.

## Figures and Tables

**Table 1:** Characterizations of polysaccharides isolated from RS, BZ, FL, and GC.

	RS-3-1	RS-3-2	BZ-3-1	BZ-3-2	BZ-3-3	FL-3-1	GC-3-1
Total sugar (%)	91.1	91.5	91.8	94.1	97.0	96.0	97.1
Protein (%)	—	—	—	—	—	—	—
Uronic acid (%)	2.2	1.1	2.6	3.5	1.4	0.9	0.8
Molecular weight	5.6 × 10^4^	5.4 × 10^4^	5.7 × 10^4^	5.6 × 10^4^	5.5 × 10^4^	5.6 × 10^4^	5.4 × 10^4^

—: not detected; RS: Radix Ginseng, Ren Shen; BZ: Rhizoma Atractylodis Macrocephalae, Bai Zhu; FL: Poria, Fu Ling; GC: Radix Glycyrrhizae, Gan Cao.

**Table 2:** Monosaccharide composition of polysaccharides Isolated form RS, BZ, FL, and GC.

	RS-3-1	RS-3-2	BZ-3-1	BZ-3-2	BZ-3-3	FL-3-1	GC-3-1
Rha (%)	10.6	—	14.1	2.6	—	0.8	1.3
Ara (%)	15.2	—	39.4	7.6	—	2.1	2.6
Fuc (%)	2.8	—	—	—	—	3.1	—
Xyl (%)	—	4.3	1.4	—	—	—	—
Man (%)	2.8	23.5	4.2	21.1	15.4	10.8	15.9
Glc (%)	52.9	72.2	22.6	62.6	84.6	71.7	76.9
Gal (%)	15.7	—	18.3	6.1	—	11.5	3.3

—: not detected; RS: Radix Ginseng, Ren Shen; BZ: Rhizoma Atractylodis Macrocephalae, Bai Zhu; FL: Poria, Fu Ling; GC: Radix Glycyrrhizae, Gan Cao.
